# Intensive leaf cooling promotes tree survival during a record heatwave

**DOI:** 10.1073/pnas.2408583121

**Published:** 2024-10-14

**Authors:** Bradley C. Posch, Susan E. Bush, Dan F. Koepke, Alexandra Schuessler, Leander L.D. Anderegg, Luiza M.T. Aparecido, Benjamin W. Blonder, Jessica S. Guo, Kelly L. Kerr, Madeline E. Moran, Hillary F. Cooper, Christopher E. Doughty, Catherine A. Gehring, Thomas G. Whitham, Gerard J. Allan, Kevin R. Hultine

**Affiliations:** ^a^Department of Research, Conservation and Collections, Desert Botanical Garden, Phoenix, AZ 85008; ^b^Department of Environmental Science, Policy and Management, University of California Berkeley, Berkeley, CA 94720; ^c^Department of Ecology, Evolution and Marine Biology, University of California, Santa Barbara, Santa Barbara, CA 93106; ^d^School of Biological Sciences, University of Utah, Salt Lake City, UT 84112; ^e^Arizona Experiment Station, University of Arizona, Tucson, AZ 85721; ^f^School of Life Sciences, Arizona State University, Tempe, AZ 85287; ^g^Department of Biological Sciences and Center for Adaptable Western Landscapes, Northern Arizona University, Flagstaff, AZ 86011; ^h^School of Informatics, Computing, and Cyber Systems, Northern Arizona University, Flagstaff, AZ 86011

**Keywords:** climate change, thermal regulation, stomatal conductance, plant hydraulics, heat tolerance

## Abstract

Increasing heatwaves are threatening forest ecosystems globally. Leaf thermal regulation and tolerance are important for plant survival during heatwaves, though the interaction between these processes and water availability is unclear. Genotypes of the widely distributed foundation tree species *Populus fremontii* were studied in a controlled common garden during a record summer heatwave—where air temperature exceeded 48 °C. When water was not limiting, all genotypes cooled leaves 2 to 5 °C below air temperatures. Homeothermic cooling was disrupted for weeks following a 72-h reduction in soil water, resulting in leaf temperatures rising 3 °C above air temperature and 1.3 °C above leaf thresholds for physiological damage, despite the water stress having little effect on leaf water potentials. Tradeoffs between leaf thermal safety and hydraulic safety emerged but, regardless of water use strategy, all genotypes experienced significant leaf mortality following water stress. Genotypes from warmer climates showed greater leaf cooling and less leaf mortality after water stress in comparison with genotypes from cooler climates. These results illustrate how brief soil water limitation disrupts leaf thermal regulation and potentially compromises plant survival during extreme heatwaves, thus providing insight into future scenarios in which ecosystems will be challenged with extreme heat and unreliable soil water access.

## Significance

High temperatures inhibit leaf function and can cause plant death. Despite ongoing increases in heatwave occurrence, understanding of how plants survive extreme heat exposure is limited. While mechanisms for both leaf heat avoidance and tolerance have been identified, it is unknown how these strategies are affected by water availability during an extended heatwave. We show that the riparian tree *Populus fremontii* is highly efficient at leaf cooling via transpiration, even when air temperature exceeds 48 °C. However, a minor disruption in soil water availability completely inhibits leaf cooling, causing leaves to exceed critical temperature thresholds. These results provide new insight into the limited capacity forest ecosystems have for cooling their canopies below critical thresholds during extreme heatwaves.

High temperature records on virtually every continent are increasingly being broken and rebroken as the incidence and intensity of extreme heatwaves continues to rise. These novel conditions are causing ambient temperatures to approach and exceed thermal thresholds for physiological damage in a wide range of plant taxa (1–3). Consequently, many plant populations are rapidly becoming maladapted to their climates, which will likely have considerable impacts on carbon storage, biogeochemical cycling, and biodiversity (4, 5). High temperature thresholds are especially important for leaves given the central role they play in CO_2_ assimilation and thus plant growth. Plants have evolved two strategies for maintaining leaf function during extreme high temperature exposure—leaf temperature regulation and leaf thermal tolerance (6). However, our understanding of how these two strategies function under extreme air temperatures (e.g., >45 °C) remains unclear, due to the difficulty inherent in either replicating such conditions in controlled environments or conducting field-based measurements during naturally occurring extreme heatwaves (7, 8). Intraspecific comparisons of genotypes could prove especially valuable for identifying evolutionary trends in leaf thermal regulation and tolerance given that tradeoffs among functional trait syndromes and strategies may be necessary to cope with heat stress. Such comparisons would thus likely highlight potential limits and opportunities for adaptation in focal species.

Maintaining a positive leaf thermal safety margin—defined as the difference between the critical temperature at which photosystem II (PSII) electron transport rapidly declines (T_crit_) and maximum leaf temperature (T_leaf_, i.e., leaf thermal safety margin = T_crit_−T_leaf_)—is likely key for maintaining leaf function during extreme heatwaves. In most heat-exposed plants T_crit_ rarely exceeds 52 °C, generally falling between 45 to 50 °C (1, 9–11). While temporal acclimation to heat exposure is common (12–14), data from wheat have shown T_crit_ eventually plateaus at high temperatures regardless of previous exposure (15). Thus, in progressively hotter environments, leaf thermal safety increasingly relies on maintaining T_leaf_ at or below air temperature (T_air_), provided air temperature does not exceed T_crit_. A primary mechanism by which plants maintain T_leaf_ below T_air_ is transpiration, with recent experiments demonstrating some plants facilitate leaf cooling by increasing stomatal conductance to water vapor (*g*_sw_) in hot conditions (7, 11, 16–18). However, this carries potential risks of hydraulic failure via reduced xylem hydraulic conductivity due to air embolism, reduced leaf turgor pressure, or both. Consequently, the extent of limited homeothermy (i.e. T_leaf_/T_air_ slope < 1) particularly under limited water availability, remains an open question (for a detailed description of the limited homeothermy hypothesis, see ref. 19). In multiple forest types, canopy T_leaf_ has been reported to regularly exceed T_air_, suggesting that limited homeothermy is not widespread, at least in cases where T_air_ remains below 35 °C (20, 21). It is plausible that tree canopies exposed to episodic heatwaves may cross tipping points unless T_leaf_ is maintained below T_crit_ (21). If novel heatwave conditions trigger widespread leaf damage, tree function and forest biogeochemical cycling may be altered across multiple scales.

**Fig. 1.**
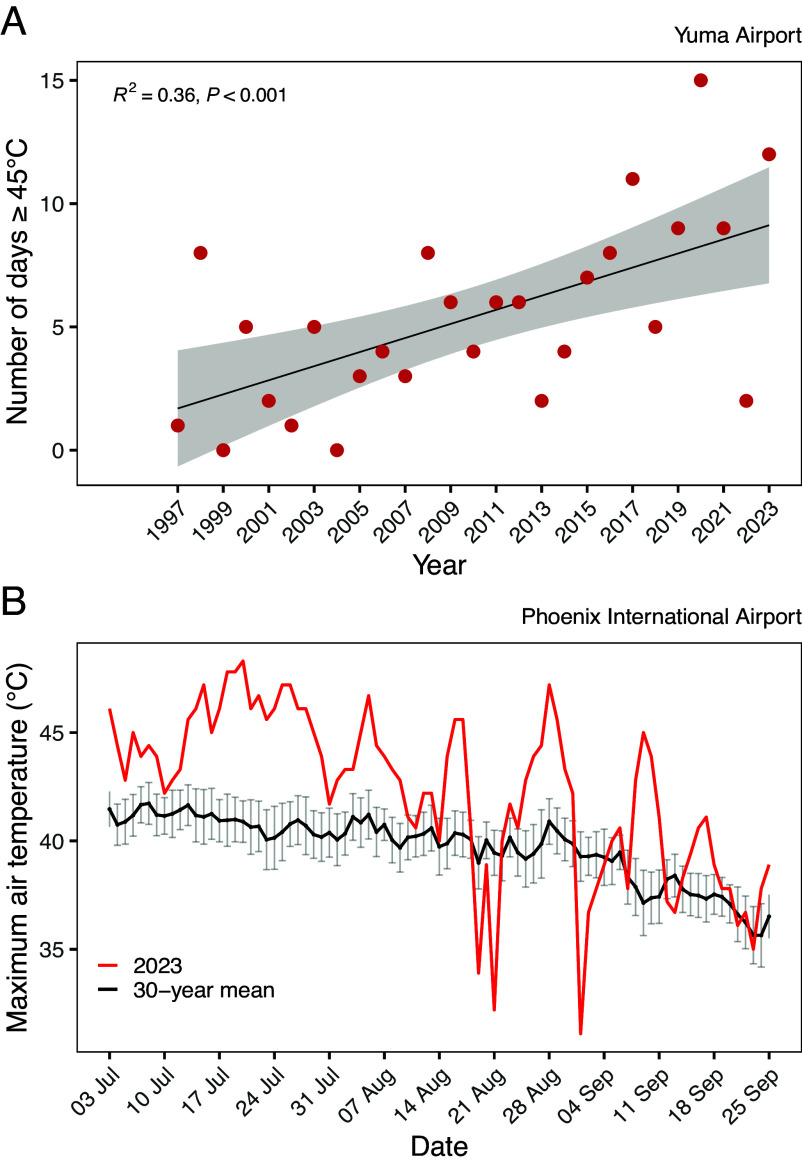
(*A*) Number of days per year when maximum air temperature reached or exceeded 45 °C between 1997 and 2023 at the Yuma airport weather station, 75 km from the lowest elevation and hottest site from which trees were sourced for this experiment. The shaded area denotes 95% CI. (*B*) Daily maximum temperature as measured at the Phoenix International Airport between July 1 and September 30, from 1993–2023. The red line denotes daily maximum temperatures recorded during this period in 2023. The black line denotes daily maximum temperatures calculated for each day as mean from 1993–2022, error bars denote SE (n = 30).

*Populus fremontii* (Wats.) is a model species for evaluating the thermal limits of broad-leaved tree taxa and investigating tradeoffs between hydraulic safety and thermal regulation. This winter deciduous species occurs in warm deserts and spans a broad climate gradient—ranging from the hottest regions in North America to locations that regularly experience spring freeze-thaw events (22)—and shows high levels of local adaptation (23, 24). The extent of climatic variation across *P. fremontii* populations provides an opportunity to assess the extent to which thermal environment influences physiological responses to heat in a single species. Populations along the warm edge of its distribution in the southwestern US, where T_air_ approaches or exceeds 50 °C and the frequency of days surpassing 45 °C is increasing (Fig. 1*A*), have experienced recent mortality surges (25). Under well-watered conditions, warm-adapted *P. fremontii* genotypes exhibit limited homeothermy on days when T_air_ and vapor pressure deficit (*D*) remain below 40 °C and 5 kPa, respectively (11). However, it remains unclear whether *P. fremontii* and other broad-leaved tree taxa can maintain T_leaf_ below thermal thresholds when T_air_ exceeds 45 °C, and to what extent this thermal regulation is related to maintaining hydraulic safety.

We evaluated leaf thermal and hydraulic safety in two-year-old *P. fremontii* saplings in an experimental common garden during the hottest summer on record, occurring in 2023, in Phoenix, AZ (26). Genotypes from four source populations were studied, spanning a 1,500m elevation gradient. The elevation gradient also corresponded to a thermal gradient; the mean daily maximum temperature at the lowest elevation was 39.6 °C, while for the highest elevation it was 32.8 °C (Table 1). Mean daily maximum T_air_ during the experiment was 42.2 °C and included a period when maximum T_air_ exceeded 45 °C for 17 consecutive days (Fig. 1*B*). The record heatwave conditions allowed us to evaluate two interrelated questions: Does thermal environment predict the capacity of trees to cool leaves below thermal thresholds during extended extreme heat exposure? And if so, does homeothermic leaf cooling during heatwaves come at the cost of greater risk of hydraulic failure when exposed to water stress?

**Table 1.** Source site name, elevation, latitude, longitude, mean daily maximum temperature (MDMT) between Jul–Sep for 1993–2022 and 2023, and mean basal stem diameter of trees in a common garden in Phoenix, AZ measured August 14, 2023

Source site	Elevation (m)	Latitude	Longitude	MDMT Jul–Sep 1993–2022 (°C ± SD)	MDMT Jul–Sep 2023 (°C ± SD)	Mean basal stem diameter (mm ± SE)
Colorado River	70	33.362	–114.698	39.6 (±2.5)	41.9 (±3.3)	25.0 (±0.8)
New River	666	33.948	–112.136	37.4 (±1.9)	38.7 (±3.2)	21.9 (±0.5)
San Pedro River	1219	31.610	–110.167	33.6 (±1.7)	34.9 (±2.4)	22.1 (±0.8)
Little Colorado River	1,521	34.961	–110.390	32.8 (±2.4)	34.3 (±3.5)	23.1 (±0.7)
Phoenix irport	339	33.428	–112.004	39.5 (±1.6)	42.2 (±3.6)	–

Numbers in parentheses represent ± the SE of the means.

Addressing these questions not only improves our understanding of tree thermal limits in the face of extreme high temperatures, but also advances our knowledge of how short-term changes in soil moisture availability can alter plant thermal regulation and vulnerability to heat stress. Considering the current American southwest megadrought (27) has coincided with increasingly hot temperatures (28), and that 30% of trees globally are threatened with extinction (29), the results of this study have implications that span from local to global scales.

## Results

### Environmental Conditions at Common Garden and Tree Source Locations.

The maximum recorded T_air_ during the experiment was 48.3 °C on July 20, 2023 (Fig. 1*A*). Between July 1 and September 30 there were 28 d where the maximum temperature was ≥45 °C, and the mean daily maximum temperature during this period was 42.2 °C. In 2023, 87% of days between July 1 and September 30 were hotter than the 30-y average (Fig. 1*B*). The maximum leaf vapor pressure deficit (*D*_leaf_) recorded during the experiment was 9.4 kPa, recorded in late August. During the hottest part of the day (2 to 5 pm) mean atmospheric vapor pressure deficit (*D*) was 7.4 kPa, with a maximum *D* of 10.3 kPa on July 20.

### Relationship Between Leaf and Air Temperature.

**Fig. 2.**
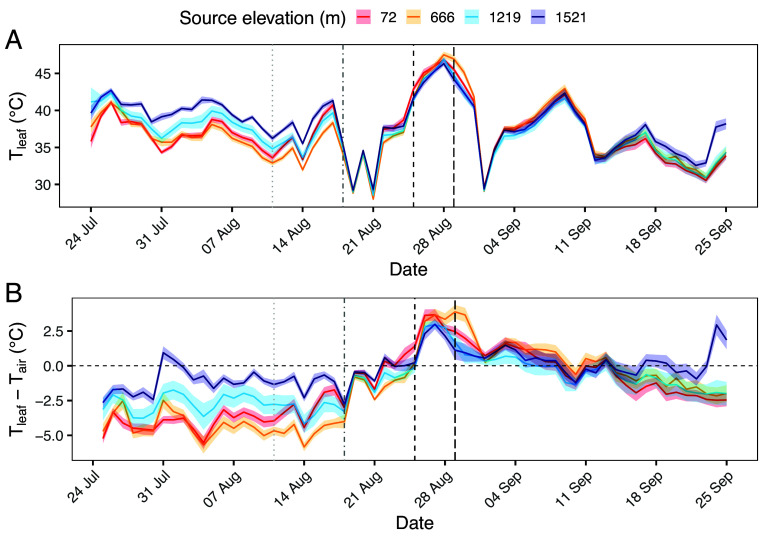
(*A*) Mean afternoon leaf temperature (T_leaf_) from July 25–September 25, 2023, in *Populus fremontii* genotypes sourced from four populations. Source location of populations ranged in elevation from 72 to 1,521 m. Mean T_leaf_ calculated as average of T_leaf_ measured at 15 min intervals between 14:00 to 18:00 each day in three pseudoreplicate leaves per tree, and three replicate trees per population. (*B*) The difference between T_leaf_ and air temperature (T_air_) was also calculated for the same period. Shaded areas around lines show 95% CI around means. Plants were irrigated for 20 min every 6 h throughout experiment, except for periods indicated by broken vertical lines: Aug 11 (light gray dotted line) irrigation decreased by 50% to 10 min every 6 h; Aug 18 (gray dash-dot line) irrigation decreased by 50% to 5 min every 6 h; and Aug 25 (black dashed line) irrigation decreased by 70% to 3 min every 12 h. Original irrigation regime was resumed from Aug 29 (black long dash line). The horizontal dashed line shows equilibrium between T_leaf_ and T_air_.

From July 25 to September 25, 2023, the mean afternoon T_leaf_ between 14:00 to 18:00 was 37.2 °C, and the highest single T_leaf_ measurement was 53.9 °C (Fig. 2*A*) during the peak of the water stress treatment. Overall, leaf temperature was positively correlated with elevation of source sites—1,521 and 1,212 m populations had significantly warmer leaves than 666 m population (*SI Appendix*, Table S1). Trees sourced from the highest elevation site (1,521 m) had the highest mean T_leaf_ (38.2 °C), while individuals from the 72 m and 666 m sites had the lowest (36.8 and 36.9 °C, respectively). From July 25 to August 11, when all plants were well watered and mean maximum daily T_air_ was 44.2 °C, T_leaf_ was almost entirely maintained below T_air_ (Fig. 2*B*), with T_leaf_ of the two lowest elevation populations averaging >4 °C cooler than T_air_. By contrast, leaves from the high elevation population averaged only 1.1 °C cooler than T_air_. However, these patterns disappeared under water restriction. When irrigation was at its lowest point (August 25–29), T_leaf_ across all genotypes averaged 3 °C warmer than T_air_. After original levels of irrigation were resumed on August 30, ΔT (i.e. T_leaf_–T_air_) for all genotypes generally declined, although T_leaf_ remained warmer than T_air_ for approximately 2 wk following the water stress treatment.

### Afternoon Stomatal Conductance.

**Fig. 3.**
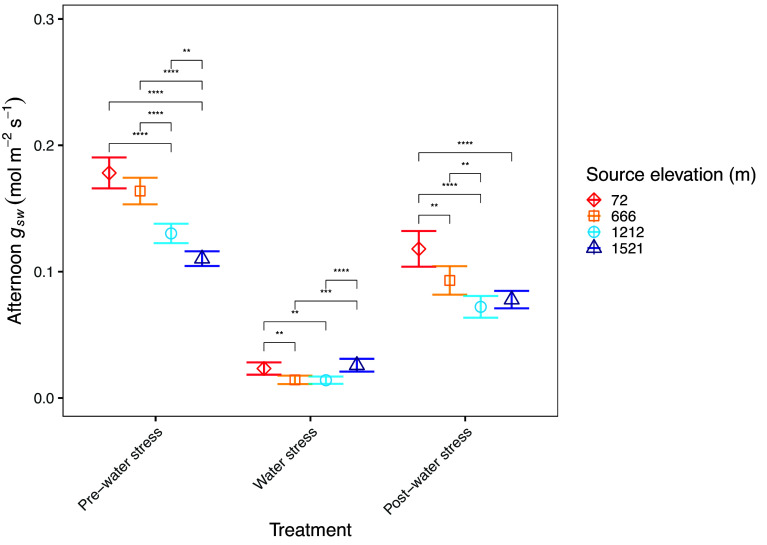
Mean afternoon leaf stomatal conductance (*g*_sw_) of *P. fremontii* trees measured between 15:00 to 16:30 at seven time points between Jul 25–Sep 25, 2023. The first four time points (Jul 25–Aug 24) are pooled and termed “pre-water stress.” The middle time point (Aug 25–Aug 28) coincided with the lowest level of irrigation and is termed “water stress.” The last two time points (Aug 29–Sep 25) are pooled and termed “post-water stress.” Source location of *P. fremontii* populations ranged in elevation from 72 to 1,521 m. Error bars represent ± SE of the means [for pre-water stress n = 20 (5 genotypes * 5 time points); for water stress n = 5 (5 genotypes); for post-water stress n = 10; (5 genotypes * 2 time points)]. The Tukey HSD test was used to analyze post hoc differences between populations at each treatment period, asterisks denote significant differences (*< 0.05; **< 0.01; ***< 0.001; ****< 0.0001).

During the pre-water stress period (four time points between Jul 25–Aug 24), low elevation genotypes exhibited a significantly higher mean *g_sw_* (Fig. 3). Specifically, the mean afternoon *g*_sw_ of the 72 m population (0.18 mol m^−2^ s^−1^; SE = ±0.006) was *c.* 40% higher than that of the 1,521 m population (0.11 mol m^−2^ s^−1^; SE = ±0.003). When considering the pre-water stress measurement timepoints individually (*n* = 4, *SI Appendix*, Fig. S1), the differences in afternoon *g*_sw_ between populations were apparent during the first two timepoints, when all trees were well watered and T_air_ reached its peak. The difference in afternoon *g_sw_* between populations disappeared; however, during the water stress period, when *g_sw_* approached zero in all trees (Fig. 3). During the post-water stress period (two time points between Aug 29–Sep 25) afternoon *g_sw_* increased for all trees, and the lowest elevation trees again had the highest *g_sw_*, though the between-sites differences in *g_sw_* were less pronounced than during the pre-water stress period (Fig. 3). Over the course of the study, a significant interaction was detected between population and irrigation treatment (*SI Appendix*, Table S1).

### Leaf Thermal Tolerance and Quantum Yield of Photosystem II.

**Fig. 4.**
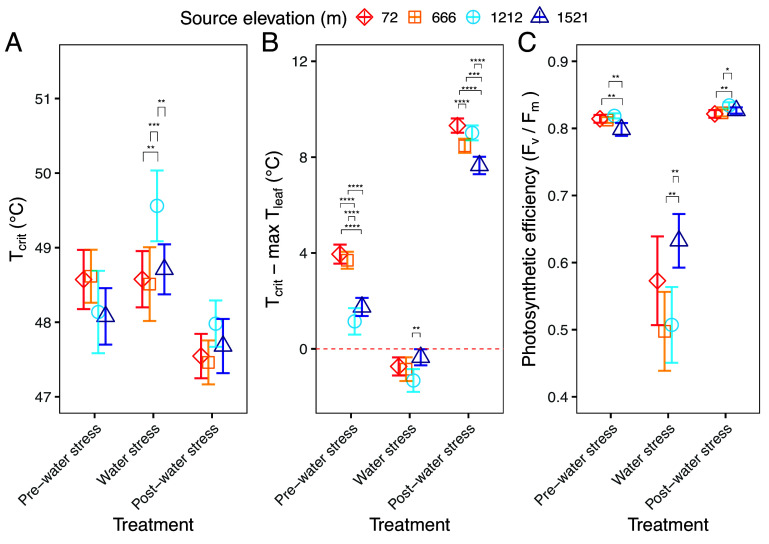
(*A*) Mean temperature at which photosystem II becomes inhibited (T_crit_); (*B*) leaf thermal safety margins (calculated as *T*_crit_—maximum T_leaf_) in relation to the point at which T_crit_ = T_leaf_ (denoted by the horizontal red dashed line); and (*C*) maximum photosystem II efficiency (F_v_/F_m_, measured at least one hour after sunset). All were measured at a single time point during each of the three experimental treatment periods: pre-water stress, water stress, and post-water stress. Error bars represent ± SE of the means (n = 5 genotypes). The Tukey HSD test was used to analyze post hoc differences between populations at each treatment period, asterisks denote significant differences (*< 0.05; **< 0.01; ***< 0.001; ****< 0.0001).

Photosynthetic thermal tolerance, measured as *T*_crit_, varied significantly with treatment period, though changes were subtle. Mean *T*_crit_ of all populations rose from 48.4 °C to 48.9 °C from pre-water stress to water stress period, before falling to 47.7 °C in the post-water stress period (Fig. 4*A*). Source elevation was not associated with variation in *T*_crit_; however, the interaction of treatment and source elevation did have a small, significant effect on *T*_crit_ (*SI Appendix*, Table S1), driven by a 1.5 °C increase in mean *T*_crit_ of the 1,212 m population during the water stress period, followed by a return to pre-water stress level during the post-water stress period.

Leaf thermal safety margins (T_crit_−T_leaf_) varied significantly across populations and treatments (Fig. 4*B*), driven predominantly by differences in leaf temperature across the populations and time points. During the pre-water stress period, the two lowest elevation populations maintained thermal safety margins close to 4 °C (e.g. their T_leaf_ was *c.* 4 °C lower than their T_crit_), while the 1,212 m population had the smallest thermal safety margin of 1.2 °C (SE = ±0.27). During the water stress period, the thermal safety margins of all populations became negative (Fig. 4*B*), meaning that mean T_leaf_ exceeded the respective T_crit_ of all populations. The 1,212 m population was again the most negatively affected, with a mean thermal safety margin of −1.3 °C (SE = ±0.24). By the end of the post-water stress period, all populations once again had positive thermal safety margins, ranging from 9.3 °C (SE = ±0.15) for the highest elevation population, to 7.7 °C (SE = ±0.18) for the lowest elevation population.

The strong decline in *g*_sw_ during the peak water stress period, which led to increased T_leaf_ (*SI Appendix*, Fig. S2) and subsequent breaching of leaf thermal safety margins, was also accompanied by a 32% decline in maximum photosynthetic quantum efficiency (measured as F_v_/F_m_) across all populations (Fig. 4*C*). During this period the high elevation population maintained the highest F_v_/F_m_ (0.63 unitless, SE = ±0.02), while the 666 m population exhibited the lowest F_v_/F_m_ (0.50, SE = ±0.03). Photosynthetic quantum efficiency returned to pre-water stress levels for all plants during the post-water stress period (Fig. 4*C*).

### Trade-off Between Leaf Thermal Safety and Plant Hydraulic Safety.

Leaf water potential measurements were collected both at pre-dawn (Ψ_pd_) and midday (Ψ_md_) for all trees at each of the seven measurement time points (*SI Appendix*, Fig. S1). There was no significant variation associated with population for either Ψ_pd_ or Ψ_md_ (*SI Appendix*, Table S2). Further, Ψ_pd_ did not significantly differ across water stress treatments. Although a significant difference in Ψ_md_ based on measurement time point was detected, the overall range in Ψ_md_ across the time points was very small, with a low value of −2.26 MPa (SE = ± 0.035) in the first measurement period (Jul 17–23) and a high value of −1.88 MPa (SE = ±0.027) during the fourth measurement period (Aug 21–27). Mean Ψ_88_ did not significantly differ among the three highest elevation populations, ranging from −2.94 to −3.04 MPa. The lowest elevation (and hottest) population was at −2.75 MPa, and therefore had the smallest HSM_K_ when using Ψ_88_ (overall mean Ψ_88_ = −2.91 MPa, SE = 0.04). Meanwhile, Ψ_TLP_ was similar across populations and time points (mean Ψ_TLP_ = −2.92, SE = ±0.002, *SI Appendix*, Table S3).

**Fig. 5.**
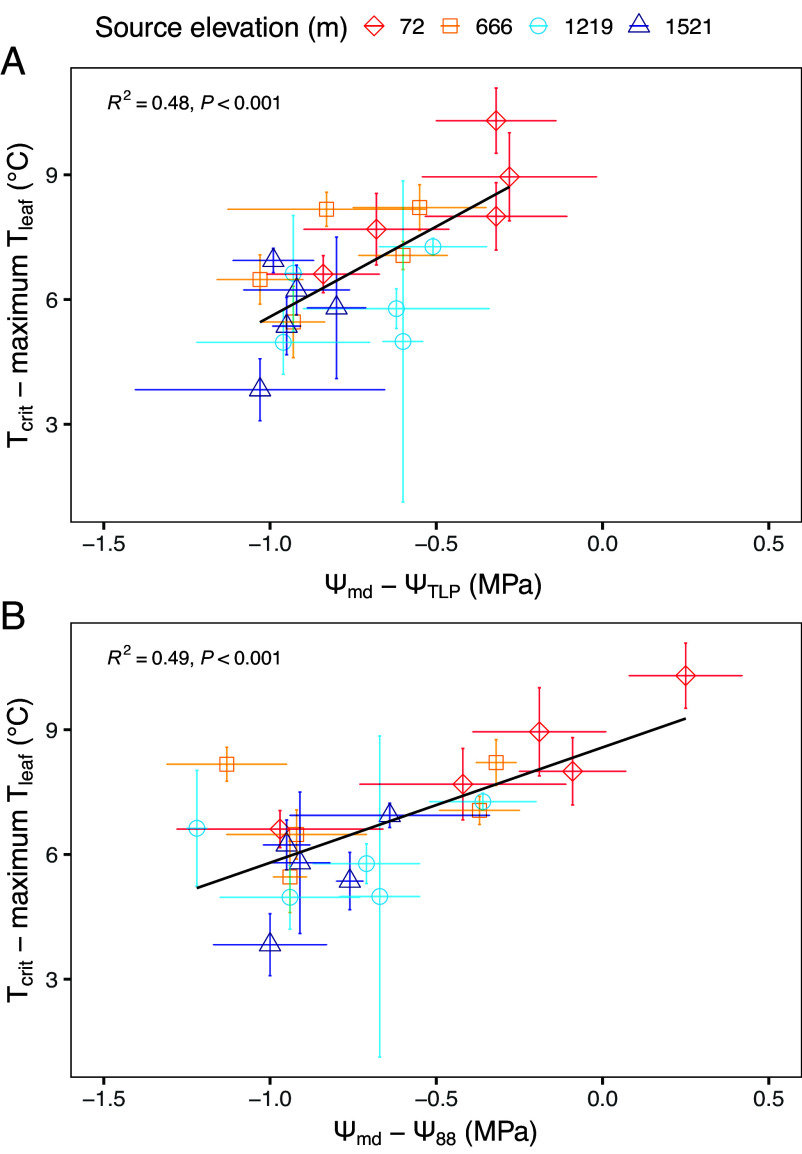
Larger thermal safety margins were associated with smaller hydraulic safety margins. (*A*) Mean hydraulic safety margins calculated using leaf turgor loss point and (*B*) stem conductance loss point (Ψ_88_) plotted against leaf thermal safety margins. Error bars represent ± SE of the means error (n = 3 individuals). The black line represents the fit from a linear model regression, and the R^2^ and *p*-value from the model are provided in the figures. All measurements collected on July 25, when plants were well watered.

We observed a significant tradeoff between hydraulic safety and thermal safety margins during the hottest day of the experiment, July 25 (Fig. 5). Larger thermal safety margins were associated with smaller hydraulic safety margins. This was true regardless of whether hydraulic safety margins were calculated based on Ψ_TLP_ or Ψ_88_. The lowest elevation population was at the furthest extreme of this relationship, maintaining the largest leaf thermal safety margins and the smallest hydraulic safety margins (Fig. 5). However, despite the clear variation in leaf thermal regulation and hydraulic strategy among populations, there was no variation detected among populations in total leaf area reduction following the water stress treatment, where mean ratio of total leaf area to stem basal area (A_l_/A_b_) fell 31% following the water stress among all populations (SE ± 0.05, *SI Appendix*, Table S4).

## Discussion

Few studies have evaluated plant thermal regulation and tolerance during naturally occurring extreme heat, and fewer still have also examined concomitant measures of hydraulic safety. Therefore, we lack understanding of how plant responses to extreme heatwaves are governed by both heat tolerance thresholds and the capacity to cool leaves via evapotranspiration, as well as how these processes are affected by disruptions in soil water supply. Leveraging record hot summer temperatures (maximum daily T_air_ reached or exceeded 45 °C for 28 d from July 1–September 30, 2023) allowed us to evaluate thermal thresholds of *P. fremontii*, a foundation tree species in riparian ecosystems distributed throughout the warm deserts of North America (22). We found that T_leaf_ of genotypes from the warmest locations was 4 to 5 °C cooler than T_air_, even when T_air_ exceeded 48 °C. However, during the warmest period of the summer, a clear tradeoff was detected between leaf thermal safety and hydraulic safety, with warm-adapted genotypes adopting greater risks of hydraulic failure to maximize leaf thermal safety. Nevertheless, a short-term, modest reduction in soil water availability—so modest it went largely undetected by predawn leaf water potential measurements—was associated with a total disruption of leaf cooling in all genotypes, resulting in *c.* two weeks where T_leaf_ exceeded T_crit_. Brief soil water supply disruption was also associated with rapid onset (e.g. within three days) of canopy dieback and stem mortality for almost all trees. Populations adapted to hotter climates achieved the greatest level of cooling and had the smallest hydraulic safety margins, suggesting they may be most vulnerable to extended disruptions in water availability during extreme heat episodes. These results indicate that leaf cooling during extreme heat, while effective, is likely a high-risk strategy given that water availability is frequently colimiting during summer heatwave exposure. It also appears more prevalent in populations adapted to very hot conditions. When considering these results alongside previous studies (14, 30), interspecific variation in leaf cooling responses under extreme heat is likely large. While we still require a greater understanding of the extent of this variation and the mechanisms driving it, based on the current results exposure to extreme heatwaves may limit both thermal and hydraulic safety envelopes in some species regardless of plant water use strategy and thus may drive selection against certain populations.

Stomatal optimization models provide a wealth of evidence that increased leaf-to-atmosphere vapor pressure deficit constrains leaf conductance and photosynthesis (31, 32). However, some recent studies challenge extant model theory, instead showing that above a certain temperature threshold *g*_sw_ increases independent of *D* in ways that enhance leaf cooling at high temperatures (17, 18, 33, 34). For instance, previous common garden studies of *P. fremontii* showed *g*_sw_ of warm-adapted genotypes increased from spring to summer, resulting in increased leaf cooling during the peak of summer heat (11, 35). In the present study, all genotypes—including those from the highest elevation where mean maximum summer temperatures only reach 33.6 °C—maintained T_leaf_ below T_crit_ under well-watered conditions, even when T_air_ exceeded 48 °C. The observed afternoon leaf cooling corresponded with leaves maintaining a relatively high *g*_sw_ despite very high levels of *D*, which peaked at a remarkable 10.3 kPa when *g*_sw_ was measured on July 20. To our knowledge, the present study is one of few (see also 7, 8) to report transpirational cooling of T_leaf_ below T_air_ in plants operating in extreme yet naturally occurring conditions of atmospheric aridity and heat, and perhaps the only to report T_leaf_ exceeding T_crit_ during an in situ heatwave.

One required resource for the transpirational cooling response is soil water access (measured via predawn leaf water potential, or Ψ_pd_) such that *g*_sw_ remains unconstrained. In the present study, no relationship was detected between watering treatment and mean Ψ_pd_. Even at the height of the water stress treatment, mean Ψ_pd_ across all genotypes remained above −0.9 MPa, well within the range typically found in groundwater-dependent vegetation (36). Despite these relatively modest changes in Ψ_pd_ during water stress, mean afternoon *g*_sw_ of all genotypes dropped 86%, and afternoon T_leaf_ warmed above T_air_ when water was temporarily withheld. This resulted in reduced maximum quantum efficiency (indicative of leaf damage) and eventual drastic leaf shedding in all genotypes. Importantly, the transition from leaves being cooler than T_air_ to warmer than T_air_ was slow to reverse after trees returned to being well-watered. Consequently, T_leaf_ hovered at or above T_air_ for more than two weeks following the water stress treatment, illustrating the legacy that transient water stress occurring during a heatwave can have on leaf thermoregulation. Populations from higher, cooler sites were also slower to return T_leaf_ below T_air_, suggesting a lack of adaptation to extreme heat may inhibit postheatwave recovery speed. A slow recovery following drought has been proposed as a potential acclimation response that may better prepare trees for subsequent stress exposure at the expense of growth (37). Given their riparian habit and heavy reliance on soil water, it is possible that cottonwoods have developed a slower return to pre-drought function to protect against further hydraulic stress, though this topic requires further investigation.

The rapid shifts from limited homeothermy to megathermy (when T_leaf_ exceeds T_air_) could help explain why, in contrast to several leaf-level studies, recent tower-based canopy temperature measurements failed to detect significant homeothermic cooling. For example, recent tower-mounted thermal sensor measurements of canopy temperature across a broad range of forest types detected no limited homeothermy behavior (20, 21). One explanation for the contrast between leaf-level measurements and canopy-scale measurements is that tower-mounted thermal sensors predominantly capture the upper canopies of mature forests where leaf water availability is constrained by limits to long-distance water transport through the xylem (38–40). Under such scenarios, hydraulic limits could reduce transpiration below the threshold necessary to evaporatively cool leaves below air temperatures. Alternatively, soil water limitations, low *D*, and low boundary layer conductance could all restrict forest canopy cooling. Another possibility is that leaf cooling responses were not triggered in previous studies where T_air_ ranged mostly between 20 to 30 °C and rarely exceeded 35 °C, a temperature regime that likely closely aligns with photosynthetic thermal optimums. The significant homeothermy observed at T_air_ greater than 45 °C suggests some plants have evolved to effect significant levels of leaf cooling at the cost of adopting a greater risk of hydraulic failure. Nevertheless, our results coincide with other recent findings that show that even modest hydraulic disruptions can greatly inhibit leaf cooling at high T_air_ (7, 14, 30). Further investigation will be required if we are to identify and ultimately predict how common leaf homeothermy is in plant taxa during episodes of extreme heat and drought. Canopy-scale T_leaf_ measurements collected at high T_air_ are needed to evaluate potential alternative water use strategies and whether plants prioritize leaf cooling over immediate carbon gain (41).

Common garden studies have yielded strong evidence that a wide range of plant taxa, including *P. fremontii*, are locally adapted to environmental conditions (42–44). In the present study, afternoon *g*_sw_ and T_leaf_ under well-watered conditions were strongly correlated with the elevation/climate of genotype source population, with the greatest leaf cooling among genotypes from the warmest locations. In July, when T_air_ peaked and plants were well watered, cooler leaves yielded larger thermal safety margins, but also generally smaller hydraulic safety margins. The tradeoff between thermal safety and hydraulic safety was not a function of T_crit_, Ψ_TLP_, or Ψ_88_, but instead was driven almost exclusively by genotypic variation in *g*_sw_ (and thus T_leaf_) and midday leaf water potential (Ψ_md_). These results suggest warm-adapted genotypes have evolved to take on greater risks of hydraulic failure to facilitate leaf cooling during heatwaves, and thus have been selected for habitats with reliable water access. Interestingly, regardless of strategy, the water stress-induced leaf and stem dieback and consequent reduction of leaf area were independent of source population. Thus, it appears the combination of transient water stress and extreme heat stress may result in the disappearance of differences in cooling-associated traits previously observed across locally adapted populations.

Virtually, every tropical and subtropical region of the globe will experience dramatic increases in heatwave intensity and frequency this century if, as predicted, global mean temperature increases by 2 °C from the late 20th to early 21st century mean (45). These novel thermal conditions may greatly reduce the hydrological niche of plant taxa that rely on maintaining high transpiration rates to cool leaves below thermal thresholds. The need for water when record high temperatures coincide with record drought could result in rapid shifts in species distributions, or the expression of hydraulic trait composition upward in elevation or latitude (46, 47). *P. fremontii* occurring along the arid lower Colorado River corridor are a textbook example of how forests are threatened by climate change (27, 48, 49). Populations along the lower Colorado River have shown remarkable capacity to cope with T_air_ approaching and even exceeding 50 °C (11), likely utilizing shallow water tables for homeothermic leaf cooling (36). However, small reductions in the water table during recent heatwave events have triggered near-complete die-offs of mature *P. fremontii* forests (11, 25, 50). The rapid disruption of leaf homeothermy detected during the 72 h water stress treatment in the present study could help explain the recent mortality surges along the lower Colorado River. Thus, *P. fremontii* may provide a window into the future of other forest ecosystems as temperatures continue to rise, and soil water access becomes less dependable.

## Materials and Methods

### Plant Sampling and Growth in Experimental Garden.

Individuals from four populations of *P. fremontii*, a riparian tree that grows in the warm deserts of North America, were sourced along a 1,450 m elevational and 6.8 °C thermal gradient in Arizona (Table 1). Trees were established from branch cuttings using methods described in previous *P. fremontii* common garden studies (44). Cuttings of approximately 0.5 to 1 cm diameter and 25 to 30 cm in length were collected from individual genotypes at the four field locations, dipped in 0.3% indole-3-butyric acid rooting hormone, and planted in 6 × 35 cm pots. After approximately nine months’ growing in a greenhouse at Northern Arizona University in Flagstaff, AZ, the trees were transported to the Desert Botanical Garden in Phoenix, AZ. In March 2022, the trees were transplanted into 56.8 L pots approximately 43 cm × 36 cm (diameter × height) in a mix of approximately 50% potting soil and 50% red lava rock. Prior to transplanting, 600 to 800 g of peat moss was added to each pot. The trees were grown in partial shade until the end of March 2023, before the pots were moved to full sun in an experimental common garden plot (33.46482, −111.94021; 380 m elevation) where they were wrapped in reflective wrap to reduce soil warming from incident sunlight and spaced 2 m apart atop weed matting. Sixty trees were used in the study, consisting of three replicate trees from each of five genotypes per population (i.e. 3 replicates × 5 genotypes × 4 populations). To maintain sufficient soil moisture akin to the typical rooting zone along riparian areas (51), the trees were initially watered every 6 h for 20 min by drip irrigation at a rate of approximately 35 L h^−1^. On August 11 irrigation was decreased to 10 min every 6 h, and then decreased again on August 18 to 5 min every 6 h, then decreased further on August 25 to 3 min every 12 h (or 8% of initial watering amount). The peak water stress treatment began at 18:00, August 25 and concluded at 06:00, August 29, when irrigation returned to 20 min every 6 h. Measurements of temperature and relative humidity from the weather station at Phoenix Sky Harbor International Airport (<8 km from the common garden) were used to calculate atmospheric vapor pressure deficit (*D*).

### Leaf Temperature.

A subset of 12 trees—three per population—had custom-built, fine-wire thermistors affixed to the abaxial side of three fully sun-exposed leaves per tree using surgical tape. Thermistors continuously measured T_leaf_ from July 25–September 25, 2023. Measurements were logged every 30 s with a CR1000 datalogger (Campbell Scientific, UT), and 15 min averages were stored.

### Stomatal Conductance.

Leaf stomatal conductance to water vapor (*g_sw_*, mol m^−2^ s^−1^) was measured using a LI-600 porometer (Li-Cor Inc., Lincoln, NE) on three recently flushed and fully expanded leaves positioned *c.* 1.5 to 2 m above the ground in each tree. Leaves of *P. fremontii* are amphistomatous (35), thus measurements were taken on both sides of each leaf and were used to calculate an average *g_sw_*. Measurements were repeated on the same branches for all 60 trees during both the morning (07:30 to 09:00) and mid-afternoon (15:00 to 16:30) for each of the seven measurement periods. Measurement periods were pooled into three time periods for analysis: “pre-drought” (Jul 25–Aug 24); “drought” (Aug 25–Aug 28); and “post-drought” (Aug 29–Sep 25). Air temperature measurements collected using the porometer were found to be within ± 1.5 °C of concurrent air temperature data from the Phoenix International Airport weather station.

### Photosynthetic Efficiency and Thermal Tolerance.

The maximum quantum efficiency of PSII was measured with a FluorPen FP 100 hand-held PAM fluorometer (Photon Systems Instruments, Drásov, Czechia). Dark-adapted leaves were exposed to an initial 0.027 mmol m^−2^ s^−1^ light pulse to determine minimum chlorophyll *a* fluorescence (F_o_), followed by a 2,400 mmol m^−2^ s^−1^ saturating light pulse to determine maximum fluorescence (F_m_). Maximum quantum efficiency was calculated as the ratio of variable chlorophyll *a* fluorescence (F_v_, calculated as F_o_−F_m_) to F_m_ (i.e., F_v_/F_m_). Measurements were collected from three leaves per tree between 22:00 to 00:00 during three of the seven measurement periods: the initial, well-watered period on August 8; the peak water stress on August 28; and the post-water stress recovery on September 17.

Leaf thermal tolerance was measured as the thermal tolerance of PSII based on F_o_, as described in Moran et al. (11). Briefly, three sun-exposed leaves per tree were collected between 08:00 to 14:00 and dark-adapted in the laboratory for a minimum of 30 min. One 5.5 mm diameter disc was taken from each leaf and placed in a 48-well Peltier heating block that was heated from 30 to 60 °C at a rate of 0.5 °C min^−1^ by a TR2000 thermoregulator (Photon Systems Instruments, Drásov, Czechia). Measurements of F_o_ were recorded every 30 s during the heating protocol with a FC800-C FluorCam (Photon Systems Instruments, Drásov, Czechia). The resulting F_o_ temperature response curve was used to find the critical temperature of PSII (T_crit_), which was calculated as the breakpoint at which F_o_ begins to rise rapidly with temperature, as described in Schreiber and Berry (52). The breakpoint of each curve was calculated using the *segmented* (version 1.6-4) R package (53). The resulting T_crit_ values were used to find leaf thermal safety margins, which were calculated as the difference between mean T_crit_ and the maximum recorded T_leaf_ for a given population during the entire experimental period.

### Leaf Water Potential.

To quantify soil water availability and maximum water stress, leaf water potentials were measured on all 60 trees during seven different measurement periods from July 25 through September 22, 2023. Four measurement periods were during the pre-water stress period, one was during the peak water stress period, and two occurred during the post-water stress period. We measured both predawn (*c*. 03:00 to 05:00) and midday (*c*. 13:00 to 15:00) minimum water potentials (Ψ_pd_ and Ψ_md_, respectively; MPa). For each measurement, a single, fully expanded leaf at mid-canopy height was cut with a sharp razor blade at the proximal end of the petiole. The excised leaves were placed in a sealed plastic bag containing a moist paper towel and stored in a dark cooler. All water potential measurements were taken within 20 min of leaf collection using a Scholander-type pressure chamber (1505XD-EXP; PMS Instruments, Albany, OR).

### Leaf Turgor Loss Point.

Leaf turgor loss point (Ψ_TLP_), the water potential at which leaves lose turgor and many physiological functions become impaired (54), has been used as a proxy for stomatal closure (55) and so was used as one of two approaches to determining the hydraulic safety margin (e.g., HSM_TLP_ = Ψ_md_−Ψ_TLP_). Measurements of Ψ_TLP_ were made at midday in July (prior to water stress) and September (following the water stress treatment). Estimates of Ψ_TLP_ were made using a vapor pressure osmometer (VAPRO 5520; Wescor, Logan, UT), which yields similar results as Ψ_TLP_ measured from standard pressure volume curves in *P. fremontii* leaves (11). We calculated Ψ_TLP_ from measured leaf osmotic potential and the equation derived from Bartlett et al. (56):

[1] $$\Psi_{TLP}\;=\;0.832\;\Psi_{\pi 100}\;–\;0.631,$$

where osmotic potential at full turgor (Ψ_π100_) was calculated using the van ‘t Hoff equation (57):

[2] $$\Psi_{\pi 100}=\;-C_{s}RT,$$

where the osmolality of a solution, or solute concentration (*C_s_*, mmol kg^−1^), is proportional to the dew point temperature depression that is measured by the osmometer (58)_,_

*R* = 8.3145 × 10^−6^ MPa kg mmol^−1^ K^−1^ is the molar gas constant, and *T* = 298.15 K. The sampling collection, rehydration, and osmometer preparation process followed the process in Moran et al. (11). Leaf Ψ_TLP_ was measured on 2 leaves per tree and 1 to 2 disks extracted from each leaf with a 7.25 mm diameter cork borer.

### Branch Hydraulic Traits.

We evaluated branch hydraulic vulnerability to cavitation to assess differences in drought tolerance among the populations. We used the difference between Ψ_md_ and 88% loss stem conductivity point (Ψ_88_), as the safety margin for hydraulic conductivity (HSM*_K_*) (59, 60). We used Ψ_88_ rather than Ψ_50_, which is approximately −1.5 to −1.6 MPa (61), because minimum water potential has previously been close to, or even more negative than, Ψ_50_, and close to full embolism at −2.1 MPa (11, 35, 62). We selected 28 to 30 cm straight branch segments to be processed for hydraulic traits (63). In September and October 2023, one branch segment per replicate was analyzed (*n* = 60). After each branch segment was cut, the proximal end was immediately placed in water. Several branches were removed in one period, and then taken to the laboratory within 15 min. The leaves were removed to decrease water loss, and the branch segments were recut under water two to three times to reduce xylem tension. Branch segments ≥35 cm long and 0.3 to 0.8 cm sapwood diameter were shipped overnight from Phoenix, AZ to the University of California, Santa Barbara for hydraulic measurements.

Branch segments were recut under water using a sharp razor blade to lengths *c.* 28 to 30 cm, longer than vessel lengths reported for *Populus* species (64, 65). The bark layer was removed from each segment *c.* 5 cm from the cut ends. The mean diameter (mm) of each segment was recorded as the average of the basal and distal cut ends (no bark included). Samples were pressure flushed with 2% potassium chloride solution filtered to 0.2 μm at 100 kPa for one hour to remove air embolisms (42). Vulnerability curves were determined on the segments using the Cavitron method, which uses a centrifuge to induce xylem tension and measure conductance simultaneously (63). Xylem pressure was first set to a reference pressure (−1 MPa) and a maximum conductance was determined. Three to five hydraulic conductance measurements were made per xylem tension level at progressively more negative xylem tensions (*c.* 0.25 MPa increments with 1 min calibration at each increment) until percent loss of conductance reached *c.* 95%. The *fitplc* (version 1.2.3) R package was used to fit vulnerability curves and determine Ψ_88_ (tension at which 88% loss of conductance occurs) using a Weibull curve (66). Hydraulic safety margins using branch hydraulic vulnerability (HSM_K_) were calculated as the difference between minimum leaf water potential and stem Ψ_88_ (e.g., HSM_K_ = Ψ_md_−Ψ_88_).

### Leaf Area to Basal Area Ratios.

Stem basal diameter was measured at the base of each tree on August 1 and September 23 using digital calipers. Basal diameter was measured at a 90° angle at two points in the stem, below any branching, with the average between the two measurements used to calculate stem basal area (A_b_). Total leaf area (A_l_) was estimated allometrically in early August prior to the peak water stress treatment, and again in late September, after the water stress treatment. Leaf area was determined by first calculating population-specific allometric relationship between the number of leaves occurring on a branch and the diameter of branches ranging in size from *c.* 2 to 13 mm (*n* = 12 to 18 branches per population for each period). Allometric estimates of leaf area were calculated by fitting a power function between the total number of leaves and branch diameter, with each regression explaining 59 to 86% of the variation in leaf number (*SI Appendix*, Table S5). The total number of leaves on each tree was calculated by measuring the diameter of each leaf-bearing branch having a proximal diameter of *c.* > 2 mm. Leaf number per tree was converted to leaf area by estimating the percentage of leaves that fell within four specific diameter size classes from 1.5 cm to >8 cm.

### Statistical Analyses.

All statistical analyses were conducted using the R statistical environment (v. 4.2.2; R core team, 2022). Linear mixed effects models were employed to analyze changes in the dependent variables *g*_sw_, T_crit_, F_v_/F_m_, thermal safety margin, and leaf water potential, using the packages “lmerTest” (67) and “emmeans” (68). To examine effects associated with the water stress treatment, “treatment” (i.e. “pre-drought,” “drought,” or “post-drought”) was a fixed term when analyzing changes in *g*_sw_, T_crit_, F_v_/F_m_, and thermal safety margin. For *g*_sw_, the “pre-water stress” treatment encompassed four data collection time points (Jul 20–21, Aug 9–10, Aug 16–17, and Aug 23–24); the “water stress” treatment was a single time point at the immediate conclusion of the three day water stress treatment (Aug 28), and the “post-water stress” period encompassed two time points (Sep 6–9, and Sep 20–22). For T_crit_, thermal safety margin, and F_v_/F_m_ each treatment corresponded to a single data collection time point. For T_crit_ and thermal safety margin “pre-water stress” = Jul 17–18; “water stress” = Aug 28–30, and “post-water stress” = Sep 21–22. For F_v_/F_m_ “pre-water stress” = Aug 8; “water stress” = Aug 28, and “post-water stress” = Sep 17. Source population, denoted as “elevation,” was also a fixed term factor in these analyses, and the interaction between “treatment” and “elevation” was also examined. The pooling of measurement time points for *g*_sw_ data was done to allow for a more direct comparison of *g*_sw_ collected immediately following water stress with *g*_sw_ collected prior to and following water stress. We also examined changes in *g*_sw_ and leaf water potential across the seven data collection time points, with the fixed effect of “treatment” replaced by the fixed effect of “measurement period.” The random term “plant id” was included in all the analyses to account for variation between individual trees. A Tukey HSD test was used to test for differences in *g*_sw_, T_crit_, leaf thermal safety margin, and F_v_/F_m_ between populations within each treatment period. Linear regression was used to test the relationship between year and the number of days ≥45 °C recorded at the Yuma airport (a weather station 75 km from our lowest elevation source population), as well as the relationship between thermal safety margin and hydraulic safety margin.

## Supplementary Material

Appendix 01 (PDF)

## Data Availability

Data and analysis code data have been deposited in cottonwood_leafcooling2023 (https://doi.org/10.5281/zenodo.11094267) (69).
