# Supplementary material for: Intensive leaf cooling promotes tree survival during a record heatwave
Source: Proc Natl Acad Sci U S A. 2024 Oct 14;121(43):e2408583121. doi: 10.1073/pnas.2408583121 (PMC11513916; doi:10.1073/pnas.2408583121)
Supplement: Supplementary file 1 — Appendix 01 (PDF) [file pnas.2408583121.sapp.pdf]

PNAS

Supporting Information for
Intensive leaf cooling promotes tree survival during a record heatwave

Bradley C. Posch[1,2,*], Susan E. Bush[1], Dan F. Koepke[1], Alexandra Schuessler[1], Leander L.D. Anderegg[3], Luiza M.T. Aparecido[4], Benjamin W. Blonder[2], Jessica S. Guo[5], Kelly L. Kerr[3], Madeline E. Moran[6], Hillary F. Cooper[7], Christopher E. Doughty[8], Catherine A. Gehring[7], Thomas G. Whitham[7], Gerard J. Allan[7] , Kevin R. Hultine[1]

[1]Department of Research, Conservation and Collections, Desert Botanical Garden, Phoenix, AZ, 85008, USA
[2]Department of Environmental Science, Policy and Management, University of California Berkeley, Berkeley, CA, 94720, USA
[3]Ecology, Evolution and Marine Biology, University of California, Santa Barbara, Santa Barbara, CA, 93106, USA
[4]School of Biological Sciences, University of Utah, Salt Lake City, UT, 84112, USA
[5]Arizona Experimental Station, College of Agriculture and Life Sciences, Tucson, AZ, 85721, USA
[6]School of Life Sciences, Arizona State University, Tempe, AZ, 85287, USA
[7]Department of Biological Sciences and Center for Adaptable Western Landscapes, Northern Arizona University, Flagstaff, AZ 86011, USA
[8]School of Informatics, Computing, and Cyber Systems, Northern Arizona University, Flagstaff, AZ 86011, USA

Correspondence: Bradley C. Posch
Email: bposch@dbg.org

This PDF file includes:

Figures S1 to S2
Tables S1 to S5

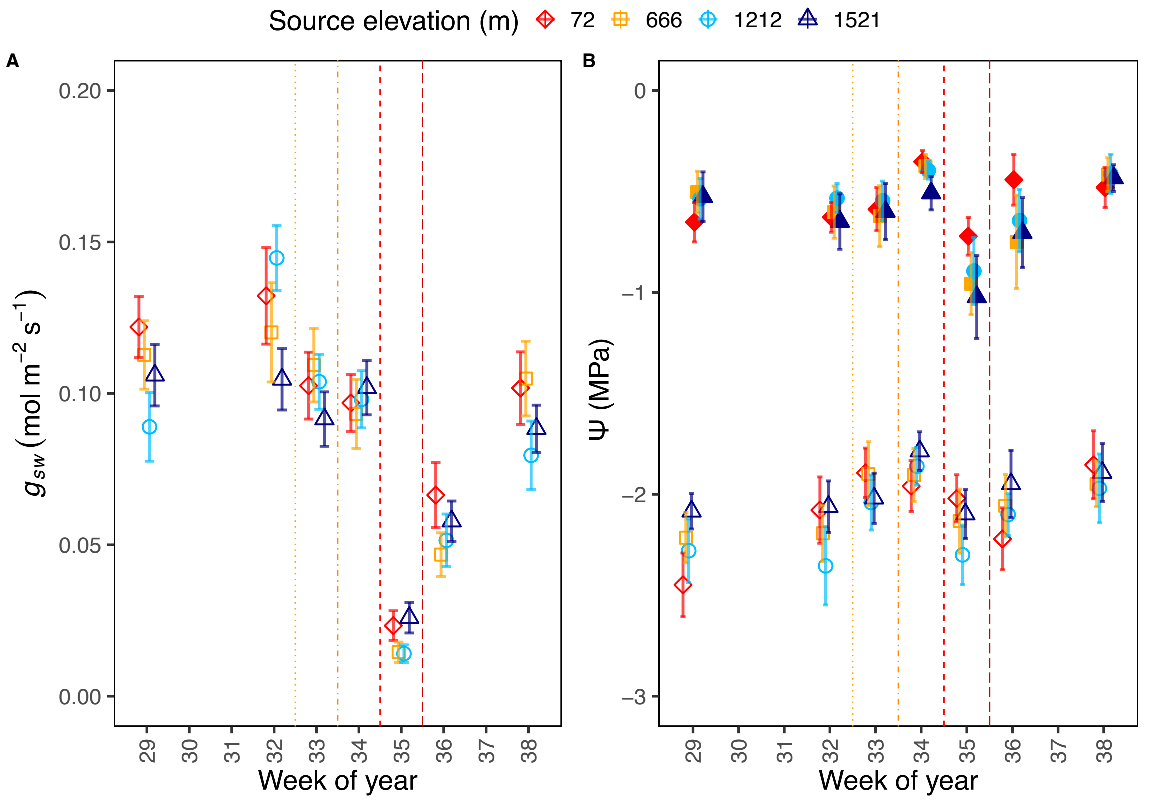

Fig. S1. Mean afternoon leaf stomatal conductance (g_{sw}), and (B) predawn (filled points) and midday (non-filled points) leaf water potential (Y) of *Populus fremontii* trees measured at seven time periods between 25th July – 25th September 2023. Elevation of source populations ranged from 72–1521m. Afternoon g_{sw} was measured between 15:00–16:30 for each time point. Plants were irrigated for 20 min every 6 h throughout experiment, except for periods indicated by colored vertical lines boxes: 11th Aug (light orange dotted line) irrigation decreased by 50% to 10 min every 6 h; 18th Aug (orange dash dot line) irrigation decreased by 50% to 5 min every 6 h; and 25th Aug (red dashed line) irrigation decreased by 70% to 3 min every 12 h. Original irrigation regime was resumed from 28th Aug (dark red long dash line). Error bars show standard error (n = 5 genotypes).

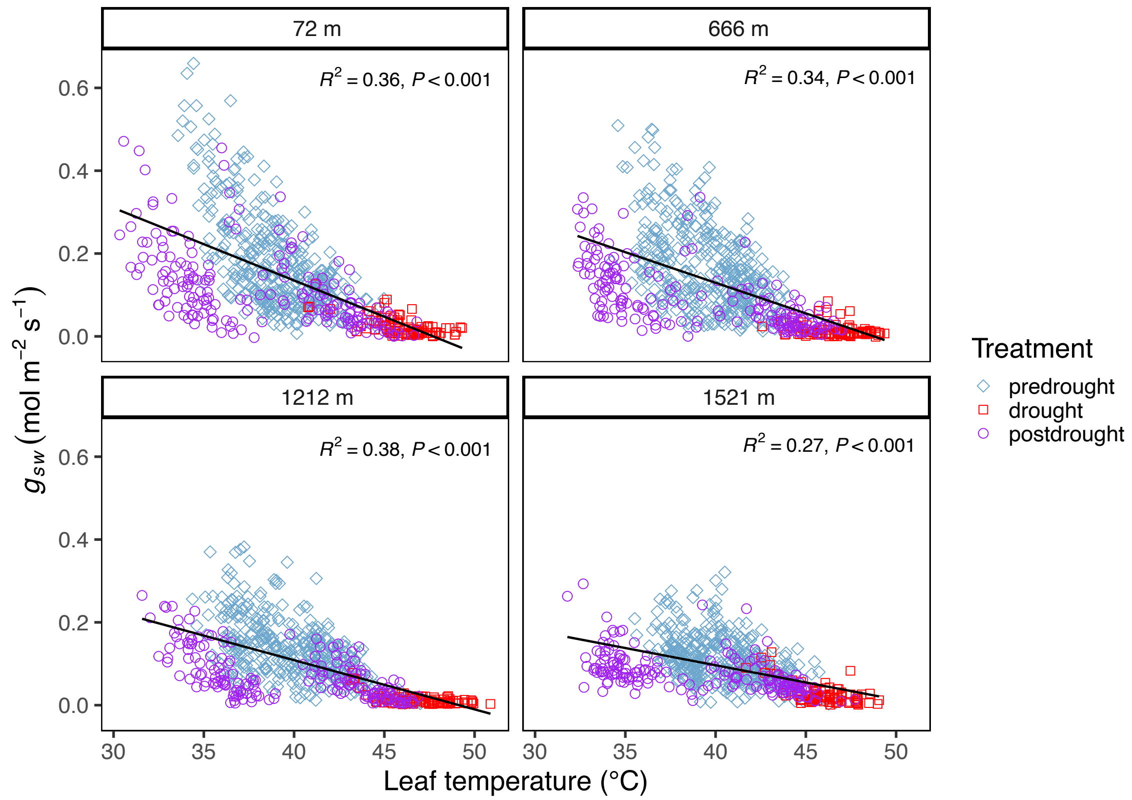

Fig. S2. Afternoon leaf stomatal conductance (g_{sw}) plotted against leaf temperature (both measured by the Licor-600 porometer) across the entirety of the experiment for four populations of *Populus fremontii* trees. Elevation of source populations ranged from 72–1521m. Afternoon g_{sw} was measured between 15:00–16:30. In 'predrought' treatment, plants were irrigated for 20 min every 6 h; in 'drought' period, irrigation was decreased by 92% to 3 min every 12 h; in 'postdrought' period, initial irrigation regime was resumed. Black lines show the fit from a linear model regression, and the R^2 and *p*-value for each respective regression are provided on the figure panels.

Table S1. Results from linear mixed models assessing the impact of source population elevation on leaf temperature (T_{leaf}), as well as analyzing the impact of elevation, treatment period (pre-water stress, water stress, and post-water stress), and their interaction on afternoon stomatal conductance (g_{sw}), leaf thermal tolerance (T_{crit}), and maximum quantum efficiency (F_v/F_m). Individual plant ID was included in all models as a random effect. When *p* values were > 0.05 effect was considered non-significant.

		T_{leaf}			g_{sw}			T_{crit}			F_v/F_m			Leaf thermal safety margin		
**Parameter**	**df**	**Den DF**	**F**	***p***	**Den DF**	**F**	***p***	**Den DF**	**F**	***p***	**Den DF**	**F**	***p***	**Den DF**	**F**	***p***
Elevation	3	19698	105.6	< 0.001	58.9	2.7	0.06	57.6	1.6	0.2	56	2.1	0.12	57.6	16.7	< 0.001
Treatment	2		-	-	2464.3	506.3	< 0.001	452.4	37.9	< 0.001	472	398.9	< 0.001	452.4	2509.2	< 0.001
Elevation * Treatment	6		-	-	2464.3	9.2	< 0.001	452.2	3.8	< 0.01	472	6.5	< 0.001	452.2	22.6	< 0.001

Table S2. Results from linear mixed models assessing the impacts of source population elevation, measurement period (seven time points between 25th July – 25th September 2023), and their interaction on afternoon stomatal conductance (g_{sw}), pre-dawn leaf water potential (Ψ_{pd}), and midday leaf water potential (Ψ_{md}). Individual plant ID was included in all models as a random effect, and water pressure chamber was also included as a random effect for Ψ_{pd} and Ψ_{md} models. When *p* values were > 0.05 effect was considered non-significant.

		g_{sw}			Ψ_{pd}			Ψ_{md}		
**Parameter**	**df**	**DenDF**	**F**	***p***	**DenDF**	**F**	***p***	**DenDF**	**F**	***p***
Elevation	3	54.74	4.67	< 0.01	56	1.8	0.16	55.44	5.63	< 0.01
Time point	6	2449.16	316.08	< 0.001	336	35.2	< 0.001	335.79	19.65	< 0.001
Elevation * Time point	18	2448.86	13.15	< 0.001	336	2.21	< 0.01	333.55	2.41	< 0.01

Table S3. Mean leaf turgor loss point (Ψ_{TLP}) measured pre-water stress and post-water stress, and percent loss of stem conductivity (Ψ_{88}) measured post-water stress in *Populus fremontii* genotypes in a common garden setting (n = 5 genotypes per population, with each genotype replicated three times). Pre-water stress measurements collection conducted on Jun 16, Jul 21, and July 27–Aug 3, 2023. Post-water stress measurements collected Sep 11–22, 2023. Different letters represent differences among populations, determined from a Tukey's HSD test. Numbers in parentheses represent the standard error of the means.

	Ψ_{TLP} (MPa)		Ψ_{88} (MPa)
**Population**	**Pre-water stress**	**Post-water stress**	**Post-water stress**
72 m	-2.93 (± 0.05)^{a}	-2.89 (± 0.04)^{ab}	-2.75 (± 0.07)^{a}
666 m	-3.01 (± 0.05)^{a}	-2.85 (± 0.05)^{ab}	-2.95 (± 0.09)^{ab}
1212 m	-3.0 (± 0.03)^{a}	-2.73 (± 0.05)^{b}	-3.04 (± 0.08)^{b}
1521 m	-3.03 (± 0.06)^{a}	-2.91 (± 0.06)^{ab}	-2.94 (± 0.05)^{ab}

Table S4. Mean leaf area to stem basal area ratios (A_l/A_b, m^2 cm^{-2}) measured in August, prior to the water stress treatment, and in September three weeks following the water stress treatment in *Populus fremontii* genotypes occurring in a common garden setting (n = 5 genotypes per population, with each genotype replicated three times). Different letters represent differences among populations, determined from a Tukey's HSD test. Numbers in parentheses represent the standard error of the means.

Population	A_l/A_b Pre-water stress	A_l/A_b Post-water stress	% change from Pre- to Post-water stress
72 m	2.93 (± 0.29)[a]	2.14 (± 0.28)[a]	-0.21 (± 0.16)[a]
666 m	1.28 (± 0.20)[b]	0.80 (± 0.13)[b]	-0.35 (±0.09)[a]
1212 m	2.11 (± 0.21)[a]	1.43 (± 0.23)[a]	-0.35 (± 0.08)[a]
1521 m	0.58 (± 0.12)[b]	0.34 (± 0.05)[b]	-0.30 (± 0.11)[a]

Table S5. Linear regression coefficients to calculate population-specific allometric relationships between number of leaves (L) and branch diameter (B_d) using the equation: $L = a \times B_d^b$.

	Pre-water stress				Post-water stress			
**Population**	***a***	***b***	***R^2***	**n**	***a***	***b***	***R^2***	**n**
72 m	1.820	2.128	0.76	13	2.595	1.884	0.59	18
666 m	1.375	2.047	0.86	12	3.591	1.762	0.77	12
1212 m	1.474	2.162	0.86	14	2.848	1.809	0.62	12
1521 m	0.697	2.140	0.83	12	0.843	1.976	0.78	13
